# The Week in the Courts

**Published:** 1910-12-17

**Authors:** 


### The Week in the Courts.

### MEDICAL PRACTITIONERS AND THE SPEED LIMIT.

At Marylebone Police Court, before Mr. Plowden, a medical practitioner was summoned for driving a motor-car at nineteen miles an hour in Regent's Park, thus exceeding the limit of twelve miles an hour. He explained to the constable that he was going to a patient who was dying, and was under the impression that under the circumstances it was legal for him to exceed the limit. The magistrate disagreed with this view of the law, but took the circumstances into consideration in fixing the penalty at 12s., including costs.

### LIBEL ACTION BY A MEDICAL OFFICER.

An action of considerable interest has been heard in the King's Bench Division of the High Court of Justice, before Mr. Justice Ridley and a special jury, by which Dr. James Thomas Macnamara, L. and L.M.R.C.S.I., L.R.C.P.Lond., medical officer under the Bermondsey Board of Guardians, to the workhouse at Ladywell, sought to recover damages for a libel alleged to have been published in the *Southwark and Bermondsey Recorder and South London Gazette.* The circumstances as related by plaintiff's counsel were as follows: In order to ensure that the consumption of brandy should be under his special supervision, Dr. Macnamara decreased the amount obtained from grocers, and had it supplied by druggists under its pharmacopœial name, *spiritus vini gulliei.* Last year an inspector drew the attention of the Local Government Board to this fact, and his report was sent by the Board to the Guardians, who asked the medical officer for an explanation. The Committee which dealt with the matter was apparently not satisfied with the explanation (some of the members complaining that they were deceived by the phrase *spiritus vini gulliei*), and expressed their dissatisfaction with the medical officer, and recommended the Guardians to inform the Local Government Board that he was not a fit and proper person to hold such an office. When the matter came before the Guardians it was adjourned to the next meeting, and in the meantime Dr. Macnamara wrote protesting that he had had no opportunity of meeting the charges, and that no complaint had ever been made against him. He then consulted Mr. Hempson, the solicitor to the Medical Defence Union, and when the matter again came before the Guardians Mr. Hempson was allowed to put questions to Dr. Macnamara, who was also questioned by members of the Committee. The result of this inquiry was that the Guardians declined to adopt the Committee's resolution, and no action was taken. In the report of the meeting at which the matter was discussed, which appeared in the local newspaper, it was stated that "Mr. Hempson . . . endeavoured to exonerate him (Dr. Macnamara) from all the charges made in the report," and there was a headline "Dr. Macnamara in Trouble." In fact, in the view of the plaintiff, the report was unfair, and made it appear that he had done something wrong, and accordingly he claimed damages. The jury returned a verdict for the plaintiff, damages £250, and judgment was entered accordingly.

